# Measurement of Histone Methylation Dynamics by One-Carbon Metabolic Isotope Labeling and High-energy Collisional Dissociation Methylation Signature Ion Detection

**DOI:** 10.1038/srep31537

**Published:** 2016-08-17

**Authors:** Hui Tang, Bing Tian, Allan R. Brasier, Lawrence C. Sowers, Kangling Zhang

**Affiliations:** 1 Department of Pharmacology & Toxicology, University of Texas Medical Branch, Galveston, Texas, 77555, USA; 2 Institute for Translational Sciences, UTMB, Galveston, Texas, 77555, USA; 3 Sealy Center for Molecular Medicine, UTMB, Galveston, Texas, 77555, USA.

## Abstract

Accumulating evidence suggests that cellular metabolites and nutrition levels control epigenetic modifications, including histone methylation. However, it is not currently possible to measure the metabolic control of histone methylation. Here we report a novel detection method to monitor methyl transfer from serine to histones through the one-carbon metabolic pathway, using stable-isotope labeling and detection of lysine methylation signature ions generated in high-energy-dissociation (HCD) tandem mass spectrometry. This method is a long-needed tool to study the metabolic control of histone methylation.

Like DNA methylation, histone methylation plays an important role in the regulation of gene expression^1^,^2^. The methylation reaction occurs via a methyltransferase with S-adenosyl-methionine (SAM) as cofactor^3^,^4^. SAM is synthesized from ATP and methionine. In addition to its production from other sources, such as cellular synthesis from L-cysteine and L-aspartate^5^, nutrition and protein degradation^6^, methionine can be synthesized via the one-carbon metabolic pathway^7^,^8^. This pathway is coupled to two metabolic cycles: the folate cycle, in which a one-carbon unit from serine or glycine is transferred to tetrahydrofolate (THF) to form 5-methylene-THF (5-meTHF), and the methionine cycle. The latter cycle produces methionine by remethylation of homocysteine from a methyl group provided by 5-meTHF, catalyzed by methionine synthase and its cofactor, vitamin B_12_. This methionine is metabolized to SAM, which serves as the rate-limiting methyl donor to major methylation substrates including DNA and histones (Fig. 1). Metabolites of folate, THF and 5-meTHF in the folate cycle are the precursors of pyrimidines and purines, which are two of the building blocks of nucleic acids (Fig. 1). Therefore, the one-carbon metabolism pathway is also linked with DNA replication during cell development.

Though histone methylation and demethylation are largely achieved by well-studied methyl writers (methyltransferases) and erasers (demethylases), the control of cofactor abundance is largely unexplored, despite the proof-of-principle that histone methylation can be detected using metabolic labeling with radioactive ^14^C-(methyl)-labeled methionine^9^. Without question, the dynamics of methylation reactions are regulated by metabolic equilibrium and cellular nutrition. Metabolic studies on the dynamics of DNA methylation have previously been conducted by stable-isotope labeling of methionine in replicating cultured cells and subsequent mass spectrometry measurements^10^. However, metabolic studies on the dynamics of histone modifications have not been performed. Here we report a novel detection method that measures the intensities of the methylation signature Ions located in the low-mass region of the HCD MS2 spectra, which avoids the need for super resolving power to separate isotopologues in the precursor ion MS1 spectra. Instead, by employing an isotope-labeling and detection strategy similar to previous approaches developed for the separation of TMT10 reporters and neutron-encoding ions (NeuCode)^11^,^12^, we show that Orbitrap instruments have sufficient resolving power to detect a one Dalton increase in heavy isotopic ions in a methylated lysine containing a cluster of ^15^N/^13^C/^2^H isotopes, allowing them to be separated and their intensities accurately measured. We demonstrate that, using this method, we are able to monitor methyl transfer from serine to histones through the one-carbon metabolic pathway.

## Results and Discussion

**Figure 1:**
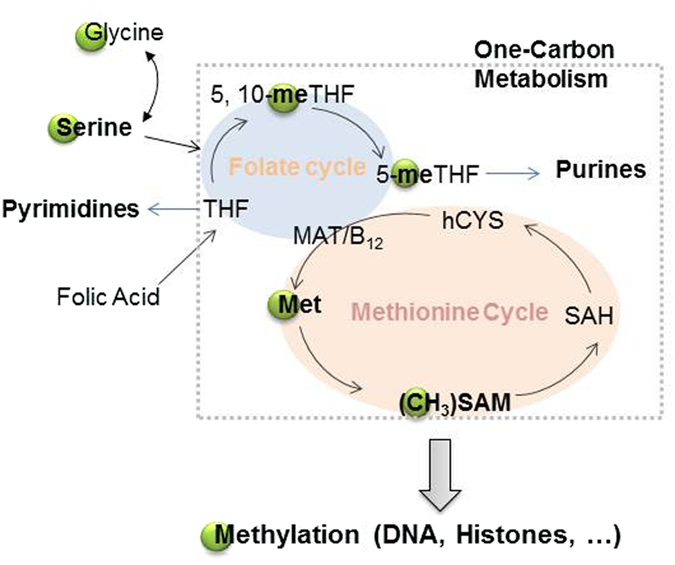
Methyl transfer from serine or glycine to DNA/histones through the one-carbon metabolic pathways. THF: tetrahydrofolate; 5, 10-meTHF: 5, 10-methylene-THF; 5-meTHF: 5-methylene-THF; MAT: methionine synthase; Met: methionine; SAM: S-adenosyl-methionine; SAH: S-adenosylhomocysteine; hCYS: homocysteine.

**Figure 2:**
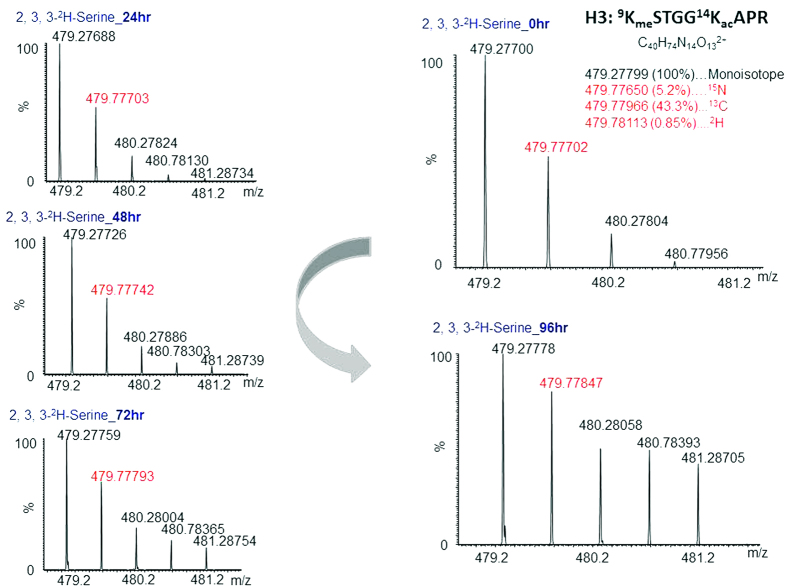
Isotopic distribution of the precursor ions in the HCD spectrum of H3 K9-mono-methyated and K14-acetylated peptides. Shown counterclockwise are the spectra of the H3 peptides digested from histones extracted from THP1 cells growing in medium containing 0.5 mM 2, 3, 3-^2^H-serine for four consecutive days in addition to the control (0, 24, 48, 72, and 96 hr). The red-numbered labeled peaks are the +1 isotopic peaks, overlapping with a cluster of three major isotopes (^15^N, ^13^C, and ^2^H) whose calculated mass values and natural occupancies (shown in nearby parentheses) are listed in the 0 hr spectrum.

The difference between ^15^N (15.0001 amu) and ^14^N (14.0031 amu), ^13^C (13.0034 amu) and ^12^C (12.0000 amu), and ^2^H (2.0141 amu) and ^1^H (1.0078 amu) is 0.9970, 1.0034, and 1.0063 amu, respectively. When being incorporated into a peptide, this tiny difference among +1 isotopes in the precursor ion and its major fragments, b and y ions, becomes almost indistinguishable and can hardly be resolved by a conventional mass spectrometer – even the Orbitrap running in a general LC-MS/MS mode. For illustration, we grew THP1 cells in medium containing 0.5 mM 2, 3, 3- ^2^H - serine for up to 96 hr and used a QExactive mass spectrometer to analyze H3 K9 mono-methylated peptides isolated after tryptic digestion of core histones. The calculated doubly-charged mono-isotopic mass of the precursor ion of this peptide was 479.27799 (100%), and the mass values of its +1 isotopic peak containing an overlapped cluster with a single isotope were 479.77650 (^15^N, 5.2%), 479.77966 (^13^C, 43.3%) and 479.78113 (^2^H, 0.85%), respectively^13^; it should be noted that the contribution from ^18^O is very small, and therefore not included in this calculation. As shown in the parentheses, ^13^C is the most naturally abundant heavy isotope in the peptide, and contributes 43.3% relative to the mono-isotope. This natural isotope distribution pattern is reflected in the spectra corresponding to histones extracted from the medium without heavy serine (Fig. 2, 0 hr). During the course of incubation with heavy serine, one-carbon ^2^H-CH_3_ and to a significantly lesser degree ^2^H_2_-CH_3_ are progressively transferred from serine to histones (Figs 1 and 2), so the proportion of ^2^H in the isotopic peaks is increasingly enriched. As shown in Fig. 2, most obviously at 96 hr, the isotopic peaks from +1 to +4 are significantly enhanced. It is not trivial to calculate the incremental intensity due to the incorporation of ^2^H from one experiment to another, nor can the accuracy of the calculation be ensured without separating the overlapping isotopic peaks. Separation of the cluster of isotopic peaks into single isotope-containing peaks requires huge resolving power. For instance, bottom-line separation of the aforementioned three +1 isotopic peaks needs at least 320,000 FWHM (Full Width at Half Maximum), based on a semi-empirical equation we used (Supplemental Calculation 1). The outcome of this calculation is fairly close to the FWTM (Full Width at 10% Maximum) value calculated by Coon’s equation derived from Gaussian peak shape modeling (Figure S1)^12^. However, even if this isotopic cluster can be separated, the portion of incorporated ^2^H from methylated lysine cannot be precisely determined from the MS1 spectrum because the peptide has one serine and two glycines (which can be synthesized from serine) that can also contribute ^2^H to the isotopic pool.

**Figure 3:**
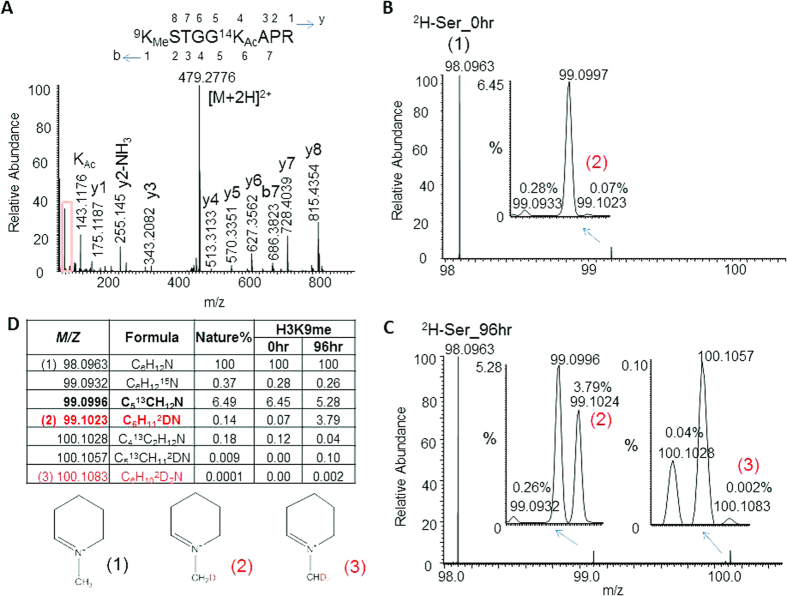
Monitoring of ^2^H-methyl in H3 K9-mono-methyated and K14-acetylated peptides by HCD immonium ion specific to mono-methylated lysine. (**A**) The full HCD tandem mass spectrum of the H3 peptide; the red-colored box includes the mono-methylated lysine-specific immonium ion at *m*/*z* 98.0963 (1). (**B**) Expanded spectrum from the red-colored boxed region in A corresponding to 0 hr of incubation with 2, 3, 3-^2^H-serine. The red-colored number (2) indicates the +1 ^2^H ion. (**C**) Expanded spectrum from the red-colored boxed region in A corresponding to 96 hr of incubation with 2, 3, 3-^2^H-serine. The red-colored number (3) indicates the +2 ^2^H ion. (**D**) A table summarizing all the isotopic ions given with their formula, calculated natural occupancy, and measured occupancy at the two time points (0 and 96 hr). Below the table are shown the hypothetical structures of the three immonium ions [(1) (2) and (3)] of mono-methylated lysine. To simplify the presentation, spectra at other time points are not shown.

The resolving power required for isotopic peak separation is proportional to the size of the molecules being separated; the smaller the molecules with close masses, the lower the resolving power needed to separate them. This concept can be applied for isolation of stable isotope-labeled methylated lysine in peptides. As we know, mono-methylated lysine generates a unique immonium ion at *m*/*z* 98.0963^14^,^15^, and its +1 isotopes with single ^15^N, ^13^C, or ^2^H have *m*/*z* of 99.0932, 99.0996, and 99.1023, respectively. Theoretically, separation of these +1 peaks only needs ~35,000 FWHM; an Orbitrap instrument is sufficient to provide this resolving power. As we expected, these ions produced from the aforementioned H3 K9 monomethylated-K14 acetylated peptide can be separated by an Orbitrap mass spectrometer with the resolution of MS2 scan set at 70,000 FWHM (Fig. 3). It is clearly seen that heavy protons were transferred from 2, 3, 3- ^2^H - serine to the CH_3_ methyl group of methylated lysine immonium ions as high as 3.7% (C_6_H_11_DN/C_6_H_12_N) at 96 hr, compared with 0.1% at 0 hr, which approximately equals the natural isotopic distribution (Fig. 3). Of course, 3.7% is itself not a large number, but considering it represents more than a 25-fold increase from the natural abundance (0.14%), it is a tremendous change that renders the tracking and quantification of isotope-labeled methylation no longer difficult. However, the change from 0.1% to 3.7% for ^2^H in (CH_3_) alone would not cause significant alteration of the isotopic distribution in the precursor ion spectrum. The greatly increased isotopic peak intensities largely arise from ^2^H-glycine (which can donate one ^2^H) converted from 2, 3, 3- ^2^H - serine for the +1 and +2 peaks, and from both ^2^H-glycine and 2, 3, 3- ^2^H - serine (which can donate three ^2^H) for the +3 and +4 peaks, as we can see in Fig. 2. Thus, the advantage of monitoring methyl-transfer via detection of the immonium ion is clearly demonstrated.

**Figure 4:**
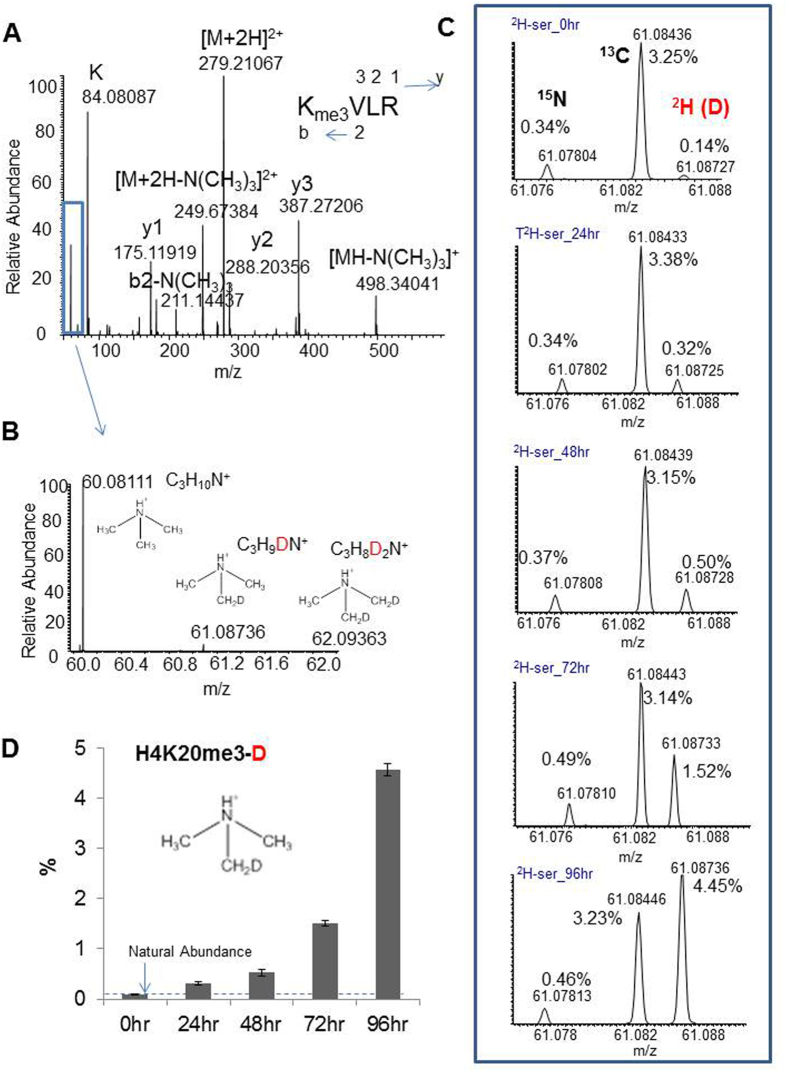
Monitoring the ^2^H-methyl in H4 K20-tri-methyated peptide by the HCD “neutral-loss” ion specific to tri-methylated lysine. (**A**) The full HCD tandem mass spectrum of the H4 peptide; the blue-colored box includes the tri-methylated lysine-specific neutral-loss ion at *m*/*z* 60.0811. (**B**) Expanded spectrum from the blue-colored boxed region in A indicates +1 and +2 ^2^H (D) ions of histones extracted from 96 hr incubation with 2, 3, 3-^2^H-serine. (**C**) Five spectra corresponding to the five time points (0, 24, 28, 72, and 96 hr) showing the expanded +1 ions containing well-resolved ^15^N, ^13^C, and ^2^H isotopes in order from left to right. (**D**) Bar graph showing the ^2^H-methyl transfer rate (relative percentage of the +1 ^2^H peak to the monoisotopic peak) at the five time points.

Due to oversaturation of the six-member ring structure by tri-methyl amine, tri-methylated lysine cannot form a mono-methylated lysine-like ammonium ion; it instead generates a prominent “neutral-loss” trimethylammonium ion [NH(CH_3_)_3_, *m*/*z* 60.0811]^14^,^15^. Like mono-methylated lysine, this small ion and its stable-isotopes +1 ions (^2^H (or D), ^13^C, and ^15^N) can be distinguished by a high-resolution Orbitrap mass spectrometer, and the ^2^H-methyl (CH_2_D) group (*m*/*z* 61.0873) transferred from 2, 3, 3-^2^H-serine to tri-methylated lysines can be monitored accordingly. Since these ions are relatively small, a resolution of 35,000 is sufficient to separate them. This concept was also tested by analyzing histone H4 K20 tri-methylation in THP1 cells that were grown in culture medium containing 0.5 mM 2, 3, 3-^2^H-serine. As shown in Fig. 4, on a four-day time scale, ^2^H-methyl is progressively transferred from ^2^H-labled serine to the H4 K4 tri-methylated peptide.

**Figure 5:**
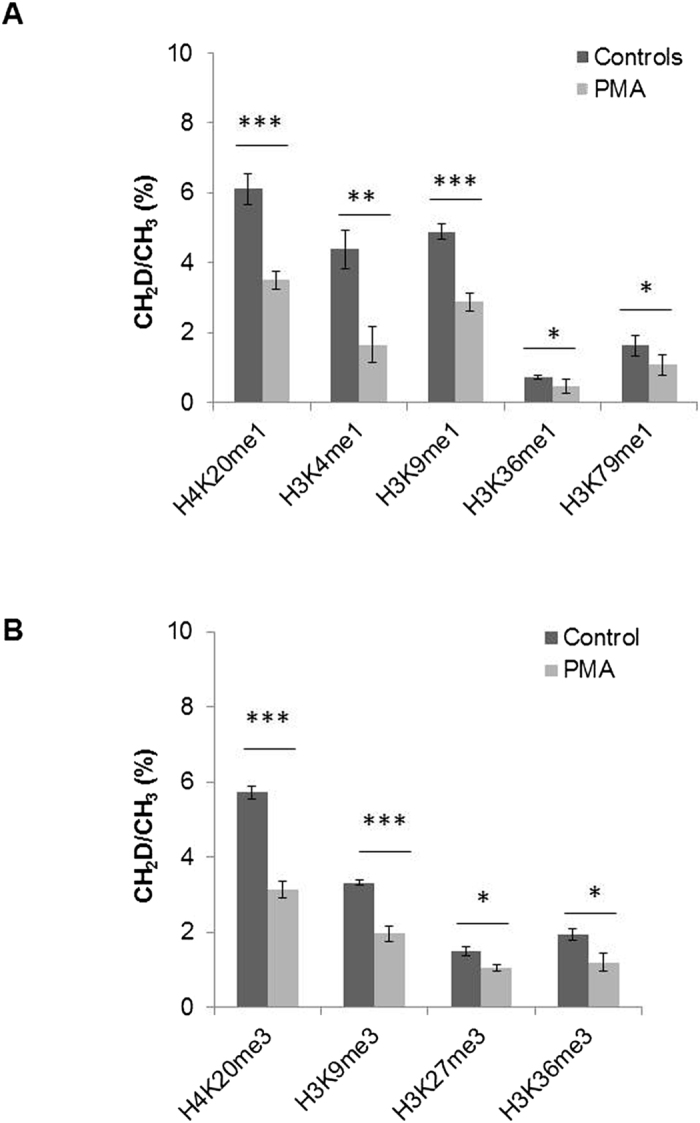
Decreased methyl-transfer rates from serine to histones in PMA-treated U937 cells. (**A**) ^2^H (**D**)-methyl transfer from 2, 3, 3-^2^H-serine to histones at the indicated mono-methylation sites. (**B**) ^2^H (**D**)-methyl transfer from 2, 3, 3-^2^H-serine to histones at the indicated tri-methylation sites. The data are averages from two biological repeats and four technical repeats. *p < 0.05; **p < 0.001; ***p < 0.0001.

To test the applicability of this method, we applied the above approach to cell differentiation/proliferation studies. In our previous proteomics analysis of protein expression pathways in human leukemic monocyte-U937 cell differentiation, down-regulation of proteins associated with the replication fork was found among the significantly disturbed pathways induced by phorbol myristate acetate (PMA), suggesting that PMA inhibits chromatin remodeling-mediated DNA replication^16^. DNA replication is coordinated with DNA synthesis at the replication fork, which requires an adequate supply of pyrimidines and purines that are the building blocks of four deoxyribonucleotide triphosphates (dNTP’s) required for DNA synthesis. Pyrimidines and purines are synthesized, respectively, from THF and 5-meTHF which are originally derived from folate acid and regulated by the serine/glycine one-carbon metabolic pathways (Fig. 1). This information garnered from previous experiments would predict an impeded one-carbon metabolism pathway in the PMA-differentiated cells that has never been explored. Further, an impeded one-carbon metabolism pathway in PMA-treated cells would result in a decrease in methionine which is synthesized by transmethylation from 5-meTHF to hCYS in the presence of methionine synthase (MAT) and cofactor vitamin B_12_ (Fig. 1). After a domino effect, SAM would be depleted and then, histone methylation is decreased. Following this lead, we could evaluate the DNA synthesis status by indirectly measuring methyl transferring rate from serine to histones (or DNA). Accordingly, we repeated our PMA-differentiation experiments by culturing U937 cells in the medium containing 0.5 mM 2, 3, 3-^2^H-serine and measuring histone methylation by ^2^H incorporation using the above-described techniques. As shown in Fig. 5A, the transmethylation rate [CH_2_D/CH_3_ (%)] at five targeted histone monomethylation sites decreased nearly 2-fold in PMA-treated cells as compared with the controls. The data also indicated that methyl-transfer reaction rates were higher for the methylation sites in protein N-termini (H3K4 & K9, and H4K20) than for sites within (or close to) the protein’s structural domain (H3K36 & K79). Similar results were obtained for trimethylation at four targeted methylation sites (H4K20 and H3K9, K27 and K36) (Fig. 5B). A decreased transmethylation rate means decreased levels of ^2^H-SAM and ^2^H-methionine generated from ^2^H-serine through the one-carbon metabolic pathway, indicating that one-carbon metabolism was impaired by PMA treatment. Taken together, our current metabolic data provide additional evidence for our previous finding that PMA down-regulates DNA replication during monocyte differentiation.

## Conclusion

We have demonstrated that methyl transfer from serine to histones through the one-carbon metabolism pathways can be monitored and quantified by measuring methylation signature ions via tandem mass spectrometry. This novel method will enable the use of mass spectrometry to study one-carbon metabolism-altered histone methylation in metabolic diseases including alcohol-mediated carcinogenesis, diabetes, Alzheimer’s disease, and tumor hypoxia^17^,^18^,^19^,^20^,^21^,^22^,^23^.

## Methods

As a proof–of-concept, we cultured THP1 cells for four consecutive days (d) in RPMI 1640 medium containing 0.5 mM 2, 3, 3,-^2^H-serine. Briefly, cells were grown in 100 × 15 mm Nunclon^®^ cell culture dishes (Thermo Scientific) with 10 mL RPMI 1640 medium containing 10% FBS and 1% antibiotics and 10 μL of the 0.5 M deuterium-labeled serine stock solution. In the control dishes (d0 or 0 hr cells), 10 μL of 0.5 M unlabeled serine stock solution was added. The medium was changed every three days. When the cells were nearly confluent, they were pelleted and core histones extracted by acid precipitation^24^. Histones were re-dissolved in 200 μL of deionized water, and 20 μL aliquots of the histone solution were digested with 1 μl of trypsin (Sigma-Aldrich) for eight hours. Histone peptides after digestion and neutralization by 1% formic acid were analyzed by LC-MS/MS on a QExactive mass spectrometer with the resolution set at either 35,000 or 70,000 FWHM. The histone peptides were separated on a 15 cm × 75 μm capillary column home-packed with 3 μm (pore size 120 Ǻ) YMC-Pack ODS-AQ reverse phase material (YMC America). A Parallel-Reaction-Monitoring (PRM) method^14^,^25^ was established to target six methylations (Table S1), including histone H3 K4, K9, K27, K36 and K79, and histone H4 K20. ^2^H-CH_3_ was monitored by the immonium ion of mono-methylated lysine at m/z 99.1024 and by the “neutral-loss” ion of tri-methylated lysine at m/z 61.0873. The dynamics of methyl transfer can be calculated based on the relative intensities after normalization against the mono-isotope immonium ion of mono-methylated lysine or the neutral-loss ion of tri-methylated lysine on the time scale of 1d, 2d, 3d and 4d. A detailed description of how to calculate methyl transfer from serine, through 1C metabolism, to the lysines in histones is provided in Supplemental Calculation 2.

For the monocyte cell differentiation study, we repeated our previous experiments on PMA-induced U937 cell differentiation^16^, with modifications. PMA is one of the well-known differentiation agents that inhibits proliferation of monocytes and converts them into M1/M2 macrophages^26^. Briefly, PMA-differentiated U937 cells were grown in RPMI 1640 medium containing 1 μM PMA and 0.5 mM 2, 3, 3,-^2^H-serine; control U937 cells were grown in the same medium, but containing only 0.5 mM 2, 3, 3,-^2^H-serine and the same amount (volume) of DMSO as was used to dissolve PMA for the differentiated cells. Both control and PMA cells were grown in triplicate under identical conditions for three days before histone isolation. Histone methylation was analyzed by the above-described PRM approach to monitor the mono-methylated lysine immonium ion and the tri-methylation neutral-loss ion.

**Data availability.** All the instrument raw data associated with this project were deposited in PeptideAtlas (ftp://PASS00876:BI5272wv@ftp.peptideatlas.org).

## Additional Information

**How to cite this article**: Tang, H. *et al*. Measurement of Histone Methylation Dynamics by One-Carbon Metabolic Isotope Labeling and High-energy Collisional Dissociation Methylation Signature Ion Detection. *Sci. Rep.* **6**, 31537; doi: 10.1038/srep31537 (2016).

## Supplementary Material

Supplementary Information
