# Supplementary material for: Measurement of Histone Methylation Dynamics by One-Carbon Metabolic Isotope Labeling and High-energy Collisional Dissociation Methylation Signature Ion Detection
Source: Sci Rep. 2016 Aug 17;6:31537. doi: 10.1038/srep31537 (PMC4987619; doi:10.1038/srep31537)
Supplement: Supplementary Information [file srep31537-s1.pdf]

Measurement of Histone Methylation Dynamics by One-Carbon Metabolic Isotope Labeling and High-energy Collisional Dissociation Methylation Signature Ion Detection

Hui Tang[1], Bing Tian[2, 3], Allan R. Brasier[2, 3], Lawrence C. Sowers[1], Kangling Zhang[1,3,] *

1. Department of Pharmacology & Toxicology, University of Texas Medical Branch, Galveston, TX
2. Institute for Translational Sciences, UTMB, Galveston, Texas, 77555
3. Sealy Center for Molecular Medicine, UTMB, Galveston, Texas, 77555

*Address correspondence to kazhang@utmb.edu

Table S1. List of precursor ions for Parallel-Reaction-Monitoring (PRM)-targeting histone mono- and tri-methylation

Precursor Ions *m/z* (Charge states)	Peptide sequence	Modification sites	Monitored ions related to methyl-transferring
265.1947 (2+)	K_{me1}VLR	H4: K20 monomethylation	98.0963 (H), 99.1023 (^{2}H or D)
359.7139 (2+)	K_{me1}QTAR	H3: K4 monomethylation	98.0963 (H), 99.1023 (^{2}H or D)
479.2774 (2+)	K_{me1}STGGK_{ac}APR	H3: K9 monomethylation	98.0963 (H), 99.1023 (^{2}H or D)
486.2853 (2+)	K_{me3}SAPATGGVK_{me1}	H3: K27 monomethylation<br>K36 trimethylation	98.0963 (H), 99.1023 (^{2}H or D)<br>60.0811 (H), 61.0873 (^{2}H or D)
675.3566 (2+)	EIAQDFK_{me1}TDLR	H3: K79 monomethylation	98.0963 (H), 99.1023 (^{2}H or D)
279.2103 (2+)	K_{me3}VLR	H4: K20 trimethylation	60.0811 (H), 61.0873 (^{2}H or D)
373.7296 (2+)	K_{me3}QTAR	H3: K4 trimethylation	Not detectible
493.2931 (2+)	K_{me3}STGGK_{ac}APR	H3: K9 trimethylation	60.0811 (H), 61.0873 (^{2}H or D)
449.9334 (3+)	SAPATGGVK_{me3}KPHR	H3: K36 trimethylation	60.0811 (H), 61.0873 (^{2}H or D)
689.3723 (2+)	EIAQDFK_{me3}TDLR	H3: K79 trimethylation	Not detectible

A. Bottom-line separation $\approx$ 2 x Full Width at Half Maximum (FWHM) (semi-empirical equation)

$$2 \times FWHM = 2 \times \frac{\frac{m}{z}}{R} = 2 \times \frac{479.3}{0.003} \approx 320{,}000;$$

in the equation, *R* is defined as the minimum *m/z* difference that can be resolved at the targeted peak.

B. Full Width at 10% Maximum (FWTM) (Coon's equation)

$$FWTM = 1.82261573 \times \frac{\frac{m}{z}}{R \times \sqrt{\frac{400}{\frac{m}{z}}}} = 1.82261573 \times \frac{479.3}{0.003 \times \sqrt{\frac{400}{479.3}}} = 318{,}755;$$

in the equation, *R* is defined as the minimum *m/z* difference that can be resolved at 400 *m/z*.

The H3K9me1 peptide with *m/z* (2+) 479.3 needs an estimated 320,000 FWHM at *m/z* 479.3 for bottom-line separation, or 318,755 FWTM at *m/z* 400 to resolve a difference of 3 mDa. When the resolution is defined at an individual mass peak, calculations for the resolving power needed for a full separation between two closed peaks with a small Δ*m/z* would be very close using the method in either **A** or **B.**

Calculation S1. Methods for the calculation of resolving power needed for separation of isotopologues.

A

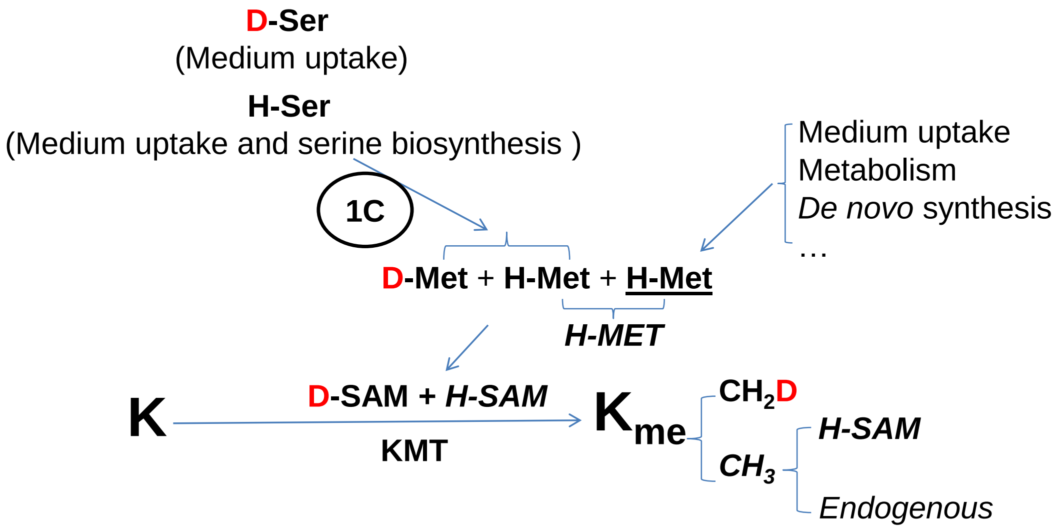

B

$$\boldsymbol{Methyl\ transfer\ from\ 1C} \approx Heavy\ atom\ (\boldsymbol{D})\ incorporation\ in\ K_{me}\ (\%) = \frac{[CH_2D]}{[CH_2D]+[CH_3]}\% \approx \frac{[CH_2D]}{[CH_3]}\%\ , when\ [CH_2D] \ll [CH_3];$$

for $\mathbf{K_{me1}}$, $$\frac{[CH_2D]}{[CH_3]}\% = \frac{I_{99.1023}}{I_{98.0963}}\%\ \text{(Immonium ion)},$$

for $\mathbf{K_{me3}}$, $$\frac{[CH_2D]}{[CH_3]}\% = \frac{I_{61.0873}}{I_{60.0811}}\%\ \text{(Neutral-loss ion)}.$$

Calculation S2. Calculation of methyl transferring from serine, through One-carbon (1C) metabolism, to histones.

A. The source from which SAM is synthesized and a cofactor for lysine methyltransferase (KMT). D-Ser: deuterium (^{2}H) labeled serine; H-Ser: unlabeled serine; D-Met: deuterium (^{2}H) labeled methionine; H-Met: unlabeled methionine from 1C metabolism; H-MET: unlabeled methionine from other sources; *H-Met*: the sum of H-Met and H-MET; D-SAM: deuterium (^{2}H) labeled S-Adenosyl methionine; *H-SAM*: unlabeled S-Adenosyl methionine; K: lysine; K_{me}: methylated lysine; CH_2D: deuterium (^{2}H) labeled methyl group; CH_3: unlabeled methyl group.

B. Calculation formula of methyl transfer from 1C to mono- and tri-methylated lysines.
